# Large-Scale Analysis of X Inactivation Variations between Primed and Naïve Human Embryonic Stem Cells

**DOI:** 10.3390/cells11111729

**Published:** 2022-05-24

**Authors:** Roni Sarel-Gallily, Nissim Benvenisty

**Affiliations:** The Azrieli Center for Stem Cells and Genetic Research, Department of Genetics, The Alexander Silberman Institute of Life Sciences, The Hebrew University of Jerusalem, Jerusalem 91904, Israel; roni.zakengalli@mail.huji.ac.il

**Keywords:** human embryonic stem cells, naive embryonic stem cells, X inactivation, large-scale analysis

## Abstract

X chromosome inactivation is a mammalian dosage compensation mechanism, where one of two X chromosomes is randomly inactivated in female cells. Previous studies have suggested that primed human embryonic stem cells (hESCs) maintain an eroded state of the X chromosome and do not express *XIST*, while in naïve transition, both *XIST* and the eroded X chromosome are reactivated. However, the pattern of chromosome X reactivation in naïve hESCs remains mainly unknown. In this study, we examine the variations in the status of X chromosome between primed and naïve hESCs by analyzing RNA sequencing samples from different studies. We show that most samples of naïve hESCs indeed reactivate *XIST* and there is an increase in gene expression levels on chromosome X. However, most of the naïve samples do not fully activate chromosome X in a uniform manner and present a distinct eroded pattern, probably as a result of *XIST* reactivation and initiation of re-inactivation of chromosome X. This large-scale analysis provides a higher-resolution description of the changes occurring in chromosome X during primed-to-naïve transition and emphasizes the importance of taking these variations into consideration when studying X inactivation in embryonic development.

## 1. Introduction

Females inherit one X chromosome from each parent, and therefore, a dosage compensation mechanism is required to balance its expression with that of males, which inherit only one maternal X chromosome [1]. There are three main dosage compensation mechanisms in nature: decreased transcription from both X chromosomes in females, present in *C. elegans* [2], two-fold increase in transcription levels from the single X chromosome in males, present in *D. melanogaster* [3] and random X inactivation, present in mammals [4]. Random X inactivation in humans occurs at the epiblast stage of embryonic development, and is suggested to be initiated by expression of *XIST*, a long non-coding RNA, which coats the X undergoing inactivation [5,6].

Human embryonic stem cells (hESCs) are derived from the inner cell mass of 5–7 days old blastocysts. The cultured hESCs are also referred to as “primed” and resemble a later, post-implementation blastocyst stage [7]. hESCs are a powerful tool extensively used in studies related to developmental processes and X chromosome inactivation during development [8,9,10]. Conversely, primed hESCs do not accurately simulate the X chromosome status during embryonic development. The majority of primed hESCs maintain an eroded state, where one X is active, and the other X is partially inactive (XaXe) [11,12,13,14,15,16,17]. Previous analysis has identified the geographical patterns of the erosion and discovered the partial inactivation in the middle of chromosome X, while the edges remain active [17,18]. In addition, unlike preimplantation blastocysts, primed hESCs do not express *XIST*. 

In contrast, cultured mouse embryonic stem cells (mESCs) can be maintained in a state similar to preimplantation blastocysts, also referred to as “naïve” state. The conversion of hPSCs from primed to naïve state can be carried out in various ways. Most protocols rely on the inhibition of MEK/Erk and GSK together with addition of LIF, and either a combination of other chemical inhibitors or overexpression of different pluripotency factors [19,20,21,22,23,24,25,26,27,28,29,30,31,32]. Naïve ESCs present a genome-wide hypomethylation and were previously reported to reactivate both *XIST* and the eroded X chromosome, resulting in two fully activated X chromosomes (XaXa) [21,33,34]. However, no global analysis regarding the variations in chromosome X between primed and naïve cells has been conducted, and the reactivation patterns remain unknown.

In this study, we describe the first large-scale analysis of X chromosome inactivation variations between primed and naïve hESCs. By analyzing a large number of RNA sequencing samples obtained from various studies, we were able to identify alterations in the expression of *XIST* and X-linked genes between primed and naïve hESCs. Intriguingly, we conclude that the reactivation of the eroded chromosome X was not full and is accompanied by further inactivation in naïve samples.

## 2. Results

### 2.1. XIST Expression in Primed-To-Naïve Transition

**Figure 1:**
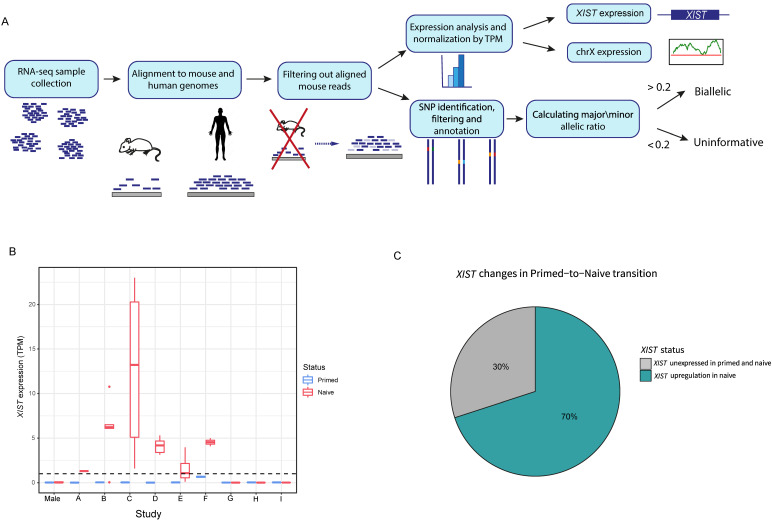
Primed and naïve hESC analysis and *XIST* expression. (**A**) Schematic representation of the pipeline of the analysis of expression of X-linked genes and their allelic expression. (**B**) *XIST* expression by TPM (transcripts per million) values in primed (blue) and naïve (red) hESCs across 9 different studies. The whiskers were calculated by the default ggplot code in R (upper whisker: min(max(x)), Q3 + 1.5 × IQR, lower whisker: max(min(x)), Q1 − 1.5 × IQR, where Q3 and Q1 are the third and first quartiles, and IQR is Q3 − Q1). The dots represent the outliers. (**C**) Distribution of *XIST* status changes across all naïve samples.

To study X inactivation variations between naïve and primed hESCs, we collected 75 samples from 11 different studies, consisting of 48 naïve samples and 27 primed samples. In these samples, we assessed three different parameters: expression of *XIST* gene, overall transcription in the X chromosome and allelic representation in chromosome X (Figure 1A). 

In all primed samples, *XIST* was not found to be expressed (TPM < 1) (Figure 1B). Out of 9 female hESC studies and 43 naïve samples, in 6 studies (labeled as studies A-F) and in 70% of all samples, there was initiation of *XIST* expression. In these samples (referred as *XIST^+^* samples), TPM values of *XIST* varied between 1.3 and 23 (Figure 1B,C). The naïve samples in studies G-I (referred as *XIST*^−^ samples) did not show *XIST* expression.

The variation in *XIST* reactivation across the analyzed samples led us to assess whether all of the studies were able to achieve naïve hESCs. In order to evaluate the “naivety” levels of the naïve samples in each study, we correlated each sample with RNA samples of human pre-implementation day 6 and day 7 epiblasts [35], focusing on 47 pre-implantation epiblast markers, obtained from a previous analysis performed on pre-implantation scRNA-seq studies [36]. As anticipated, all naïve samples showed higher correlation levels than the primed samples from the same study, including *XIST^−^* studies (Figure S1A). Interestingly, *XIST^+^* samples show higher correlation with pre-implantation epiblasts than *XIST*^−^ samples (Figure S1B).

### 2.2. Chromosome X Genes and Allelic Expression in Naïve Samples

**Figure 2:**
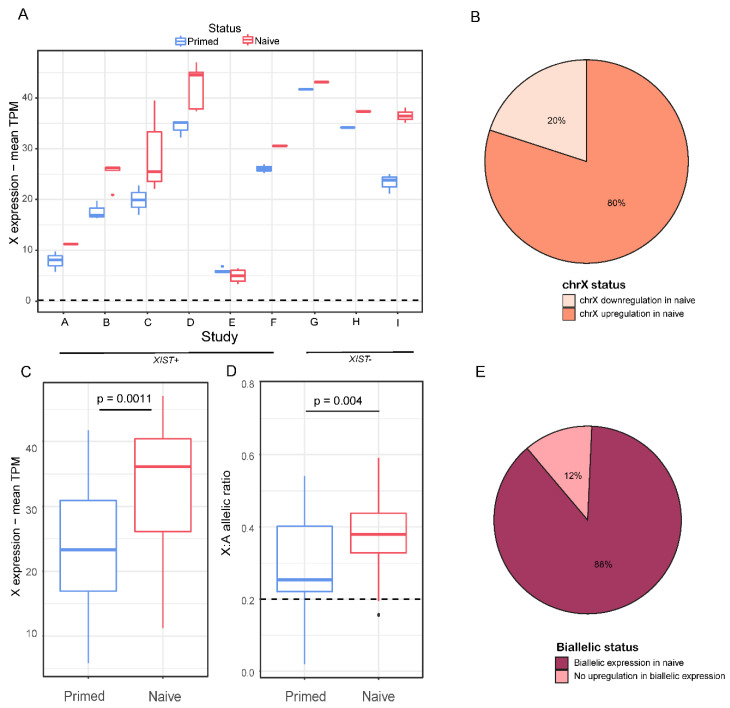
Changes in gene and allelic expression in chromosome X. (**A**) Average TPM expression of genes in chromosome X of primed (blue) and naïve (red) hESCs across 9 different studies. Whiskers and dots were calculated as mentioned in the legend to Figure 1. (**B**) Distribution of X-linked gene expression variations between naïve samples and their primed counterparts. (**C**) Average TPM expression of genes on chromosome X of primed (blue) and naïve (red) hESCs. *p*-value was calculated by a paired *t*-test. (**D**) Average X:autosomes allelic ratio of primed and naïve cells. *p*-value was calculated by a paired *t*-test. (**E**) Distribution of allelic expression variation across all naïve samples.

Next, we aimed to examine whether the entire chromosome X was reactivated in the naïve samples. We compared expression of the genes on the X chromosome in each naïve sample to the median expression of the primed sample of the same study. In almost all of the studies and in 80% of the samples, the naïve samples had higher levels of expression of X-linked genes than in their primed counterpart cells (Figure 2A,B). When averaging all of the naïve samples vs. all of the primed samples, naïve samples showed significantly higher levels of expression of X-linked genes (*p*-value < 0.01, Figure 2C). 

For the analysis of allelic expression in primed and naïve cells, we identified single nucleotide variants (SNPs) in the X-linked genes and calculated the allelic ratio (see Experimental Procedure). We addressed genes with a ratio above 0.2 as biallelic, and genes with a ratio below 0.2 as uninformative (we could not address them as monoallelic because the low ratio might derive from lack of coverage, or the absence of polymorphism in a given sample) (Figure 1A). Naïve samples demonstrated a significantly higher allelic ratio, indicating more biallelic expression than their primed counterparts (Figure 2D). Most of the studies and 88% of the samples showed an increase in the allelic ratio (Figure 2E and Figure S2A). Overall, the majority of the naïve samples displayed upregulation in the expression of X-linked genes and in biallelic representation. Additionally, we aimed to check the expression pattern of X-linked genes in primed hESCs. We normalized the TPM values of each gene in each primed sample to the median of the male hESC samples and created a moving average plot of the median along the X chromosome (Figure S2B), verifying that an eroded state (XaXe) was kept in the primed samples. In addition, we wanted to check whether *XIST* was expressed in a monoallelic or a biallelic fashion in the naïve hESCs samples. Unfortunately, only 15 samples had sufficient coverage of SNP areas (data not shown). Out of the 15 samples, 14 samples had a biallelic *XIST* expression, and 1 sample was borderline. Thus, *XIST* is expressed in a biallelic fashion in the naïve cells.

### 2.3. Geographical Pattern of Expression of X-Linked Genes and Their Allelic Representation

**Figure 3:**
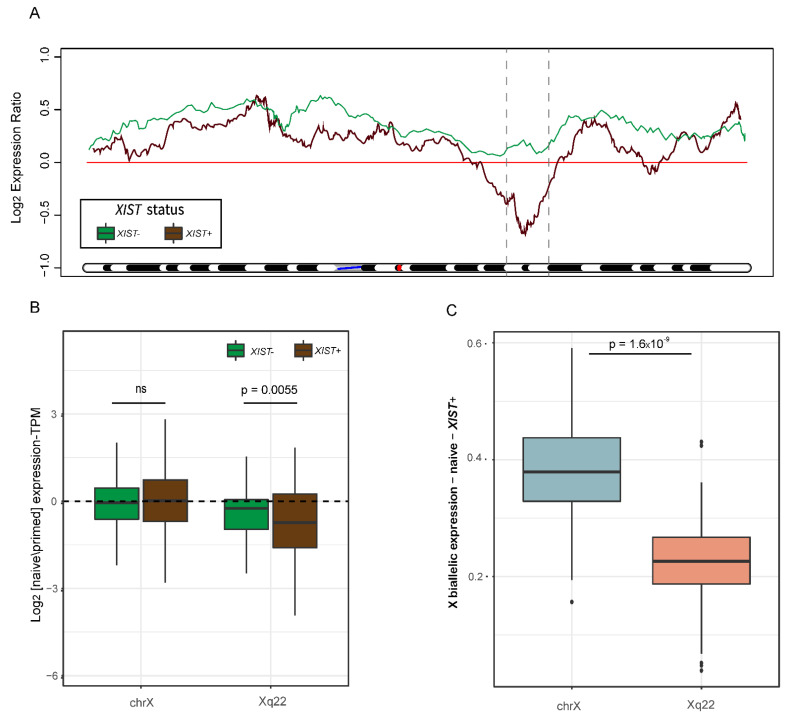
Geographical distribution of gene and allelic expression along chromosome X. (**A**) Moving average plot of median log_2_ gene expression naïve/primed ratio along chromosome X, separated into *XIST^+^* (brown) and *XIST*^−^ (green) samples. The blue line marks the location of the centromere, and the red line marks the location of *XIST.* The dashed lines define Xq22. (**B**) Chromosome X gene expression differences between *XIST^+^* and *XIST*^−^ samples in the entire X chromosome and in Xq22 area. *p*-value was calculated by a paired *t*-test. (**C**) X:autosomes allelic ratio in the entire chromosome X (light blue) versus Xq22 region (pink) in *XIST^+^* samples. *p*-value was calculated by a paired *t*-test.

Our next aim was to identify whether the activation of chromosome X is uniform in the naïve cells, or whether it is locus-specific. We thus normalized the TPM values of each gene in each naïve sample to the median of the primed samples and created a moving average plot along the X chromosome. Remarkably, the samples that activated expression of *XIST* in the naïve cells (*XIST^+^* samples) did not reactivate chromosome X in a uniform way. While the distal regions of chromosome X showed a higher expression, a clear reduction in expression in the middle of the chromosome (downstream to *XIST* gene) was observed, especially in the Xq22 area (Figure 3A and Figure S3A). In contrast, samples which did not express *XIST* did not demonstrate this specific, regional expression pattern (Figure 3A).

In addition, we compared the levels of expression of genes located at Xq22 to all genes on chromosome X in *XIST*^−^ and *XIST^+^* samples. While there was no considerable difference in the overall chromosome X gene expression levels, *XIST^+^* samples had significantly lower levels of expression in the Xq22 area (Figure 3B). Furthermore, the allelic expression ratio in *XIST^+^* was significantly decreased in Xq22 in comparison to the allelic expression ratio in all of chromosome X, indicating less biallelic expression in this area (Figure 3C).

A recent study has attempted to obtain random X inactivation in hESCs [24]. This study suggested that the reprogrammed naïve hESC populations are heterogeneous and, based on several markers, divided the naïve cells into two populations: naïve HT (which will be referred to as “high-naïve”) and naïve LT (which will be referred to as “low-naïve”). The heterogeneity and differences between high- and low-naïve hESCs were attributed to the incomplete blockage of autocrine FGF signaling, specifically FGF2. It was suggested that due to this incomplete blockage, low-naïve hESCs have low levels of FGF4 and high levels of FGF2, while high-naïve hESCs (which were able to maintain full blockage) had lower levels of FGF2 and higher levels of FGF4 [37,38]. Furthermore, it was shown that high-naïve hESCs were indeed able to initiate random X inactivation upon transitioning to primed state and these cells have closer resemblance to preimplantation epiblasts as compared to low-naïve hESCs. 

We analyzed 20 additional RNA-seq samples derived in this study, which were composed of four different cell lines, and for each cell line, we compared the high-naïve samples to the low-naïve samples. While all naïve samples expressed *XIST* (TPM > 1), most high-naïve samples had higher *XIST* expression levels than the low-naïve samples (Figure S3B). When studying the geographical patterns of gene expression along the X chromosome, high-naïve samples (each normalized to the median of their corresponding low-naïve samples) demonstrated a similar pattern to the *XIST^+^* samples (Figure 3A and Figure S3A), with a reduction in expression in the middle of the chromosome, especially around Xq22 (Figure S3C). In addition, high-naïve samples demonstrated significantly higher correlation with the epiblast markers previously mentioned (Figure S3D) [35,36].

Next, we calculated the ratio between FGF4 and FGF2 expression in each of the samples in our study. We found that while almost all samples showed an increase in FGF4/FGF2 ratio, as expected (Figure S3E), *XIST^+^* samples had a relatively higher FGF4/FGF2 ratio in comparison with *XIST*^−^ samples (Figure S3F).

## 3. Discussion

Random X inactivation is a well-known dosage compensation mechanism, present in most female mammals, and initiated at early embryonic developmental stages. Embryonic stem cells derived from human blastocysts resemble post-implementation blastocysts, and usually do not express *XIST* and do not have fully active (XaXa) or fully inactive (XaXi) X chromosomes, but an eroded state (XaXe). Reprogramming hPSCs into a naïve state, which resembles that of pre-implementation epiblasts in various aspects/features [21,39], has been suggested to be a valuable resource in studying early developmental stages. Naïve ESCs have been previously reported to have two active X chromosomes and biallelic expression of *XIST* long non-coding RNA gene. 

Here, we aimed to perform large-scale analysis of RNA sequencing data of primed and naïve hESCs from a variety of studies, in order to further understand the X reactivation process and associated patterns. We examined the differences between primed and naïve states in three main categories: expression of *XIST* gene, overall transcription of the X chromosome and chromosome X allelic representation (Figure 1A). As expected, we discovered that most naïve hESC samples upregulated *XIST* expression (Figure 1B,C). When calculating the correlation of expression of epiblast markers between the naive hESC samples and pre-implementation epiblasts, all naïve samples had higher correlation than the primed samples of each study (Figure S1A). The reactivation of *XIST* was most likely obtained due to the hypomethylation of its promoter, a part of the genome-wide hypomethylation in naïve hESCs [40]. However, *XIST^+^* samples had a higher naive/primed correlation-to-epiblast ratio than *XIST*^−^ samples. This result might indicate a higher state of naivety in *XIST^+^* samples (Figure S1B), and this is further supported by the higher FGF4/FGF2 expression ratio found in those samples (Figure S3F). As predicted, naïve hESCs had increased expression levels (with the exception of certain samples, which were all a part of one study) and a higher allelic ratio of X-linked genes (Figure 2A–E and Figure S2) than their primed equivalents. The typical hypomethylated state of naïve cells is presumably the cause for the rise in X-linked gene expression levels, and thus provides further evidence for X chromosome reactivation in naïve hESCs. 

Surprisingly, we did not observe full reactivation of the eroded X chromosome. When comparing the naïve *XIST^+^* samples to the primed samples, reactivation was observed in the distal edges of the chromosome, while a clear reduction in expression was detected in the middle of the chromosome and specifically in the Xq22 area (Figure 3A and Figure S3A). These trends of expression were repeated in the analysis of high-naïve normalized to low-naïve samples, along with higher *XIST* expression and higher correlation with epiblasts (Figure S3B–D). This unique pattern of erosion, which occurred in *XIST^+^* samples, together with the lower gene expression levels (Figure 3B) and decreased biallelic representation (Figure 3C) in this area, may suggest that chromosome X did not attain a fully activated state. Our presumption is that following the hypomethylation of the *XIST* promoter, *XIST* was activated and initiated chromosome X inactivation downstream of its location.

The inactivation localization may also be due to the high concentration of LINE-1 (L1) elements [41]. L1 elements are mammalian-specific non-LTR retrotransposons, which are mostly inactive due to truncation in the 5’ end. L1 elements are relatively abundant in chromosome X (in comparison to autosomes). They are hypothesized to be “way stations” in the process of X inactivation spreading. Previous regional analyses of chromosome X revealed that the distribution of L1 elements across the chromosome is not uniform, but instead L1 elements are clustered in specific areas, among them Xq22 [42]. The clustering of L1 elements correlates with the decrease and increase in gene expression in *XIST^+^* samples, e.g., the L1-rich areas, such as Xq13-Xq21, Xq27 and especially Xq22, have lower expression levels in naïve cells. These correlations further support our hypothesis that due to the activation of *XIST,* X inactivation was initiated and started spreading through the L1 “way stations”.

Overall, we have conducted a large-scale comparison of X-linked gene expression between primed and naïve hESCs and shed light on chromosome X dynamics in this transition. Our analysis presents a detailed, geographic profile of the X chromosome, and hints at the possibility of a specific mode of X inactivation in naïve cells, and thus provides an alternative hypothesis concerning the perception of X activation status in naïve hESCs.

## 4. Experimental Procedure

### 4.1. Sample Collection

FASTQ files of the majority of RNA sequencing samples were obtained from the SRA database (http://www.ncbi.nlm.nih.gov/sra) (accessed on 4 February 2021) [43]. Samples from An et al. (2020) were obtained from GSA database (http://bigd.big.ac.cn/gsa) (accessed on 18 July 2021) [44]. 

When selecting samples for the analyses presented in this study, our first barrier was the sex of the hESCs; in order to study X inactivation, we needed to exclude studies performed only in male cell lines. Next, we left out certain female hESC studies due to quality and coverage issues—during our data processing, we carried out several quality checks, both for gene expression levels and for allelic expression. Samples that did not pass the quality thresholds defined in our pipeline or did not have enough coverage for the stage of SNP analysis (for the allelic expression analyses) were filtered out.

Sample information can be found in Supplementary Table S1. 

### 4.2. Bulk RNA Gene and Allelic Expression Analysis

Most of the studies used in this article grew hESCs on top of mouse embryonic feeder cells (MEFs). It has been shown that many DNA and RNA samples derived from cells grown on mouse feeder cells are contaminated with reads originated in mouse [45]. In order to filter out the mouse reads from the RNA sequencing samples, FASTQ files were aligned to mouse GRCm38 and human h38 genomes using STAR aligner v2.7.6a [46], followed by filtering out reads that were mapped to the mouse genome using XenofilteR software [47]. On average, around 10% of the reads were filtered out from each sample. Next, the filtered BAM files underwent two procedures in parallel: derivation of count tables for gene expression analysis and creation of variant tables for allelic expression analysis. Count tables were generated using the *FeatureCounts* tool [48]. The variant calling procedure was performed according to the pipeline described in the Lezmi and Benvenisty paper [49], with several alterations. Briefly, using the filtered BAM files, variants were called using GATK tools: *MarkDuplicates, SplitNCigarReads, AddOrReplaceReadGroups, BaseCalibrator, ApplyBQSR,* and *HaplotypeCaller* using recommended parameters. Hard filtering of variants and exclusion of variants that did not pass the filtering and variants under 10 reads were performed using GATK’s *VariantFiltration* and BCFtools *view* tools. Annotation of variants to the GRCh38 BED exome file and establishment of variant tables was carried out using BCFtools’ *annotate* and *query* tools. 

### 4.3. Gene Expression TPM Calculation and Normalization

For establishment of normalized TPM values for each gene in each sample, a matrix containing the raw data reads of all naïve samples and a matrix containing the raw data reads of all primed samples were created. TPM was calculated by dividing the reads for each gene by the gene length, followed by division by the sum of all reads and multiplying the result by 10^6^. The expression threshold for a gene was determined as TPM > 1. For moving average plotting and geographic analysis, non-expressed genes and the top 10% most highly variable genes were removed. 

### 4.4. Pseudo-Bulk RNA Analysis of Human Epiblasts

The analysis and comparison to epiblast markers was carried out as previously described [50]. Briefly, samples from Petropoulos et al. (2016) were downloaded and aligned to h38 human genome. The 35 female cells that were determined as pre-implantation epiblasts [36] were merged to form one pseudo-bulk pre-implementation epiblast sample. The reads were normalized to TPM as mentioned above, followed by a pairwise Pearson correlation analysis of each naïve sample from each study, separately from the epiblast sample, based on 47 previously established [36] epiblast markers. Sample information can be found in Supplementary Table S1.

### 4.5. Quantifying Naïve: Primed Moving Average Plots

In order to quantify the variation between naïve and primed samples, a previously published method was utilized [51], with several modifications. For each study, the primed samples served as a baseline for calculating median TPM levels for each gene. Each naïve sample served as a test sample, and the TPM level of each gene in each sample was divided by the primed TPM median. The Log_2_ of the fold changes was then plotted in a moving average plot, drawn across chromosome X only, with a window of 50 genes. For creating a single graph of all samples, a median of all of the samples in each study was calculated and normalized as mentioned above. Next, medians of all medians of *XIST^+^* samples and *XIST*^−^ samples were created separately. 

### 4.6. Quantifying X: Autosomes Allelic Ratio

To assess the allelic expression across the X chromosome and compare it to naïve and primed samples, the X: autosomes allelic ratio was measured, as previously described [17]. Briefly, we calculated the minor/major ratio of each SNP. For normalization of SNPs in each chromosome, we divided the sum of biallelic SNPs by the number of genes on said chromosome (aka “normalized SNPs”, NS). For the X: autosome allelic ratio, we calculated the following ratio: NS (chromosome X)/mean (NS (autosomes)).

### 4.7. Statistical Methods

The statistical tests used in this study include Student’s *T*-test, Mann–Whitney non-parametric test and Pearson correlation test. The relevant method is mentioned in the corresponding figure legends.

## Supplementary Materials

The following supporting information can be downloaded at: https://www.mdpi.com/article/10.3390/cells11111729/s1, Figure S1: Correlation to epiblast samples; Figure S2: Allelic ratio of primed and naïve samples and primed geographical chromosome X expression; Figure S3: Geographical chromosome X expression, epiblast correlation, *XIST* expression and FGF ratios; Table S1: Sample table.

## References

[1] Disteche C.M. (2012). Dosage Compensation of the Sex Chromosomes. Annu. Rev. Genet..

[2] Ercan S., Lieb J.D.C. (2009). Elegans Dosage Compensation: A Window into Mechanisms of Domain-Scale Gene Regulation. Chromosome Res..

[3] Deng X., Hiatt J.B., Nguyen D.K., Ercan S., Sturgill D., Hillier L.W., Schlesinger F., Davis C.A., Reinke V.J., Gingeras T.R. (2011). Evidence for Compensatory Upregulation of Expressed X-Linked Genes in Mammals, Caenorhabditis Elegans and Drosophila Melanogaster. Nat. Genet..

[4] Nora E.P., Heard E. (2009). X Chromosome Inactivation: When Dosage Counts. Cell.

[5] Heard E., Disteche C.M. (2006). Dosage Compensation in Mammals: Fine-Tuning the Expression of the X Chromosome. Genes Dev..

[6] Brown C.J., Hendrich B.D., Rupert J.L., Lafrenière R.G., Xing Y., Lawrence J., Willard H.F. (1992). The Human XIST Gene: Analysis of a 17 Kb Inactive X-Specific RNA That Contains Conserved Repeats and Is Highly Localized within the Nucleus. Cell.

[7] De Los Angeles A., Ferrari F., Xi R., Fujiwara Y., Benvenisty N., Deng H., Hochedlinger K., Jaenisch R., Lee S., Leitch H.G. (2015). Hallmarks of Pluripotency. Nature.

[8] Hoffman L.M., Hall L., Batten J.L., Young H., Pardasani D., Baetge E.E., Lawrence J., Carpenter M.K. (2005). X-Inactivation Status Varies in Human Embryonic Stem Cell Lines. Stem Cells.

[9] Xie P., Ouyang Q., Leng L., Hu L., Cheng D., Tan Y., Lu G., Lin G. (2016). The Dynamic Changes of X Chromosome Inactivation during Early Culture of Human Embryonic Stem Cells. Stem Cell Res..

[10] Dvash T., Lavon N., Fan G. (2010). Variations of X Chromosome Inactivation Occur in Early Passages of Female Human Embryonic Stem Cells. PLoS ONE.

[11] Vallot C., Ouimette J.F., Makhlouf M., Féraud O., Pontis J., Côme J., Martinat C., Bennaceur-Griscelli A., Lalande M., Rougeulle C. (2015). Erosion of X Chromosome Inactivation in Human Pluripotent Cells Initiates with XACT Coating and Depends on a Specific Heterochromatin Landscape. Cell Stem Cell.

[12] Hall L.L., Byron M., Butler J., Becker K.A., Nelson A., Amit M., Itskovitz-Eldor J., Stein J., Stein G., Ware C. (2008). X-Inactivation Reveals Epigenetic Anomalies in Most HESC but Identifies Sublines That Initiate as Expected. J. Cell. Physiol..

[13] Shen Y., Matsuno Y., Fouse S.D., Rao N., Root S., Xu R., Pellegrini M., Riggs A.D., Fan G. (2008). X-Inactivation in Female Human Embryonic Stem Cells Is in a Nonrandom Pattern and Prone to Epigenetic Alterations. Proc. Natl. Acad. Sci. USA.

[14] Silva S.S., Rowntree R.K., Mekhoubad S., Lee J.T. (2008). X-Chromosome Inactivation and Epigenetic Fluidity in Human Embryonic Stem Cells. Proc. Natl. Acad. Sci. USA.

[15] Vallot C., Ouimette J.F., Rougeulle C. (2016). Establishment of X Chromosome Inactivation and Epigenomic Features of the Inactive X Depend on Cellular Contexts. BioEssays.

[16] Patel S., Bonora G., Sahakyan A., Kim R., Chronis C., Langerman J., Fitz-Gibbon S., Rubbi L., Skelton R.J.P., Ardehali R. (2017). Human Embryonic Stem Cells Do Not Change Their X Inactivation Status during Differentiation. Cell Rep..

[17] Bar S., Seaton L.R., Weissbein U., Eldar-Geva T., Benvenisty N. (2019). Global Characterization of X Chromosome Inactivation in Human Pluripotent Stem Cells. Cell Rep..

[18] Bruck T., Benvenisty N. (2011). Meta-Analysis of the Heterogeneity of X Chromosome Inactivation in Human Pluripotent Stem Cells. Stem Cell Res..

[19] Takashima Y., Guo G., Loos R., Nichols J., Ficz G., Krueger F., Oxley D., Santos F., Clarke J., Mansfield W. (2014). Resetting Transcription Factor Control Circuitry toward Ground-State Pluripotency in Human. Cell.

[20] Di Stefano B., Ueda M., Sabri S., Brumbaugh J., Huebner A.J., Sahakyan A., Clement K., Clowers K.J., Erickson A.R., Shioda K. (2018). Reduced MEK Inhibition Preserves Genomic Stability in Naive Human Embryonic Stem Cells. Nat. Methods.

[21] Theunissen T.W., Friedli M., He Y., Planet E., O’Neil R.C., Markoulaki S., Pontis J., Wang H., Iouranova A., Imbeault M. (2016). Molecular Criteria for Defining the Naive Human Pluripotent State. Cell Stem Cell.

[22] Park T.S., Zimmerlin L., Evans-Moses R., Thomas J., Huo J.S., Kanherkar R., He A., Ruzgar N., Grebe R., Bhutto I. (2020). Vascular Progenitors Generated from Tankyrase Inhibitor-Regulated Naïve Diabetic Human IPSC Potentiate Efficient Revascularization of Ischemic Retina. Nat. Commun..

[23] Hu Z., Li H., Jiang H., Ren Y., Yu X., Qiu J., Stablewski A.B., Zhang B., Buck M.J., Feng J. (2020). Transient Inhibition of MTOR in Human Pluripotent Stem Cells Enables Robust Formation of Mouse-Human Chimeric Embryos. Sci. Adv..

[24] An C., Feng G., Zhang J., Cao S., Wang Y., Wang N., Lu F., Zhou Q., Wang H. (2020). Overcoming Autocrine FGF Signaling-Induced Heterogeneity in Naive Human ESCs Enables Modeling of Random X Chromosome Inactivation. Cell Stem Cell.

[25] Gafni O., Weinberger L., Mansour A.A., Manor Y.S., Chomsky E., Ben-Yosef D., Kalma Y., Viukov S., Maza I., Zviran A. (2013). Derivation of Novel Human Ground State Naive Pluripotent Stem Cells. Nature.

[26] Yilmaz A., Benvenisty N. (2019). Defining Human Pluripotency. Cell Stem Cell.

[27] Chan Y.S., Göke J., Ng J.H., Lu X., Gonzales K.A.U., Tan C.P., Tng W.Q., Hong Z.Z., Lim Y.S., Ng H.H. (2013). Induction of a Human Pluripotent State with Distinct Regulatory Circuitry That Resembles Preimplantation Epiblast. Cell Stem Cell.

[28] Sperber H., Mathieu J., Wang Y., Ferreccio A., Hesson J., Xu Z., Fischer K.A., Devi A., Detraux D., Gu H. (2015). The Metabolome Regulates the Epigenetic Landscape during Naive-to-Primed Human Embryonic Stem Cell Transition. Nat. Cell Biol..

[29] Ji X., Dadon D.B., Powell B.E., Fan Z.P., Borges-Rivera D., Shachar S., Weintraub A.S., Hnisz D., Pegoraro G., Lee T.I. (2016). 3D Chromosome Regulatory Landscape of Human Pluripotent Cells. Cell Stem Cell.

[30] Guo G., von Meyenn F., Rostovskaya M., Clarke J., Dietmann S., Baker D., Sahakyan A., Myers S., Bertone P., Reik W. (2017). Epigenetic Resetting of Human Pluripotency. Development.

[31] Sahakyan A., Kim R., Chronis C., Sabri S., Bonora G., Theunissen T.W., Kuoy E., Langerman J., Clark A.T., Jaenisch R. (2017). Human Naïve Pluripotent Stem Cells Model X-Chromosome Dampening and X-Inactivation. Cell Stem Cell.

[32] Collier A.J., Panula S.P., Schell J.P., Chovanec P., Plaza Reyes A., Petropoulos S., Corcoran A.E., Walker R., Douagi I., Lanner F. (2017). Comprehensive Cell Surface Protein Profiling Identifies Specific Markers of Human Naive and Primed Pluripotent States. Cell Stem Cell.

[33] Liu X., Nefzger C.M., Rossello F.J., Chen J., Knaupp A.S., Firas J., Ford E., Pflueger J., Paynter J.M., Chy H.S. (2017). Comprehensive Characterization of Distinct States of Human Naive Pluripotency Generated by Reprogramming. Nat. Methods.

[34] Sagi I., Benvenisty N. (2016). Stem Cells: Aspiring to Naivety. Nature.

[35] Petropoulos S., Edsgärd D., Reinius B., Deng Q., Panula S.P., Codeluppi S., Plaza Reyes A., Linnarsson S., Sandberg R., Lanner F. (2016). Single-Cell RNA-Seq Reveals Lineage and X Chromosome Dynamics in Human Preimplantation Embryos. Cell.

[36] Stirparo G.G., Boroviak T., Guo G., Nichols J., Smith A., Bertone P. (2018). Integrated Analysis of Single-Cell Embryo Data Yields a Unified Transcriptome Signature for the Human Preimplantation Epiblast. Development.

[37] Krawchuk D., Honma-Yamanaka N., Anani S., Yamanaka Y. (2013). FGF4 Is a Limiting Factor Controlling the Proportions of Primitive Endoderm and Epiblast in the ICM of the Mouse Blastocyst. Dev. Biol..

[38] Ying Q.L., Wray J., Nichols J., Batlle-Morera L., Doble B., Woodgett J., Cohen P., Smith A. (2008). The Ground State of Embryonic Stem Cell Self-Renewal. Nature.

[39] Huang K., Maruyama T., Fan G. (2014). The Naive State of Human Pluripotent Stem Cells: A Synthesis of Stem Cell and Preimplantation Embryo Transcriptome Analyses. Cell Stem Cell.

[40] Pastor W.A., Chen D., Liu W., Kim R., Sahakyan A., Lukianchikov A., Plath K., Jacobsen S.E., Clark A.T. (2016). Naive Human Pluripotent Cells Feature a Methylation Landscape Devoid of Blastocyst or Germline Memory. Cell Stem Cell.

[41] Lyon M.F. (2003). The Lyon and the LINE Hypothesis. Semin. Cell Dev. Biol..

[42] Bailey J.A., Carrel L., Chakravarti A., Eichler E.E. (2000). Molecular Evidence for a Relationship between LINE-1 Elements and X Chromosome Inactivation: The Lyon Repeat Hypothesis. Proc. Natl. Acad. Sci. USA.

[43] Agarwala R., Barrett T., Beck J., Benson D.A., Bollin C., Bolton E., Bourexis D., Brister J.R., Bryant S.H., Canese K. (2016). Database Resources of the National Center for Biotechnology Information. Nucleic Acids Res..

[44] Wang Y., Song F., Zhu J., Zhang S., Yang Y., Chen T., Tang B., Dong L., Ding N., Zhang Q. (2017). GSA: Genome Sequence Archive. Genom. Proteom. Bioinform..

[45] Wan H., Fu R., Tong M., Wang Y., Wang L., Wang S., Zhang Y., Li W., Wang X.J., Feng G. (2022). Influence of Feeder Cells on Transcriptomic Analysis of Pluripotent Stem Cells. Cell Prolif..

[46] Dobin A., Davis C.A., Schlesinger F., Drenkow J., Zaleski C., Jha S., Batut P., Chaisson M., Gingeras T.R. (2013). STAR: Ultrafast Universal RNA-Seq Aligner. Bioinformatics.

[47] Kluin R.J.C., Kemper K., Kuilman T., de Ruiter J.R., Iyer V., Forment J.V., Cornelissen-Steijger P., de Rink I., ter Brugge P., Song J.Y. (2018). XenofilteR: Computational Deconvolution of Mouse and Human Reads in Tumor Xenograft Sequence Data. BMC Bioinform..

[48] Liao Y., Smyth G.K., Shi W. (2014). FeatureCounts: An Efficient General Purpose Program for Assigning Sequence Reads to Genomic Features. Bioinformatics.

[49] Lezmi E., Benvenisty N. (2021). Identification of Cancer-Related Mutations in Human Pluripotent Stem Cells Using RNA-Seq Analysis. Nat. Protoc..

[50] Keshet G., Benvenisty N. (2021). Large-Scale Analysis of Imprinting in Naive Human Pluripotent Stem Cells Reveals Recurrent Aberrations and a Potential Link to FGF Signaling. Stem Cell Rep..

[51] Weissbein U., Schachter M., Egli D., Benvenisty N. (2016). Analysis of Chromosomal Aberrations and Recombination by Allelic Bias in RNA-Seq. Nat. Commun..

