# Supplementary figures and images for: Large-Scale Analysis of X Inactivation Variations between Primed and Naïve Human Embryonic Stem Cells
Source: Cells. 2022 May 24;11(11):1729. doi: 10.3390/cells11111729 (PMC9179337; doi:10.3390/cells11111729)

A

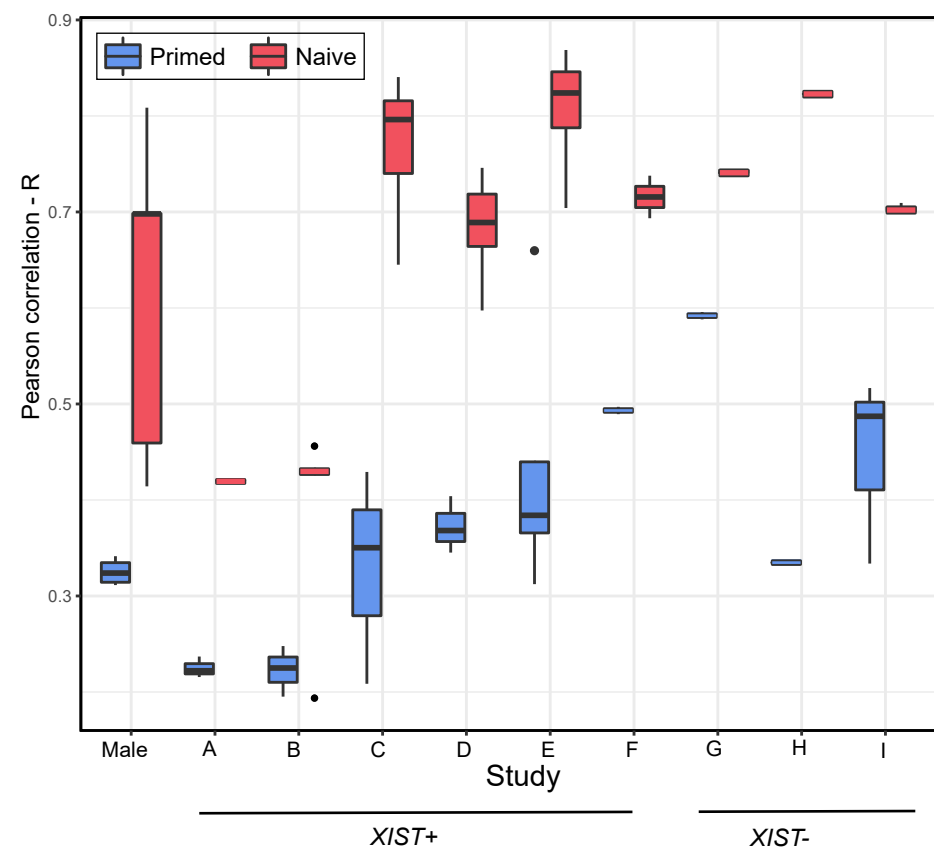

B

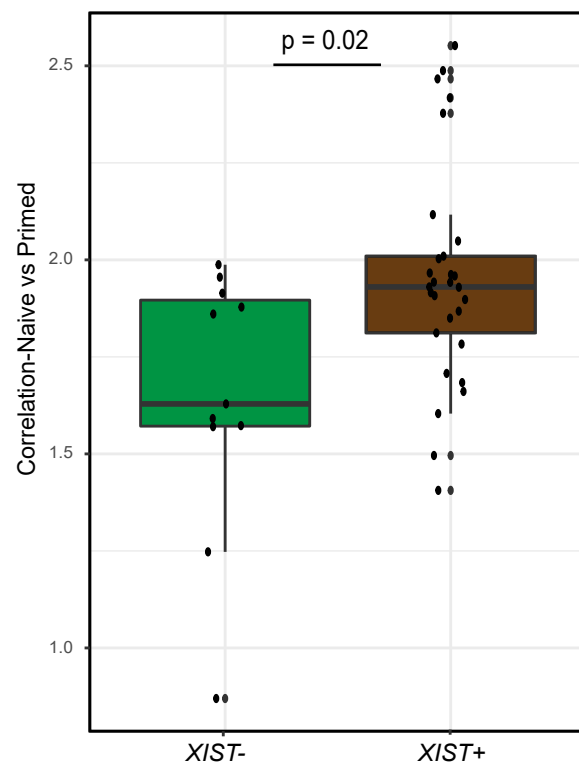

Supplement: Supplementary file 1 [file cells-11-01729-s001.zip › Figure S1.pdf]

A

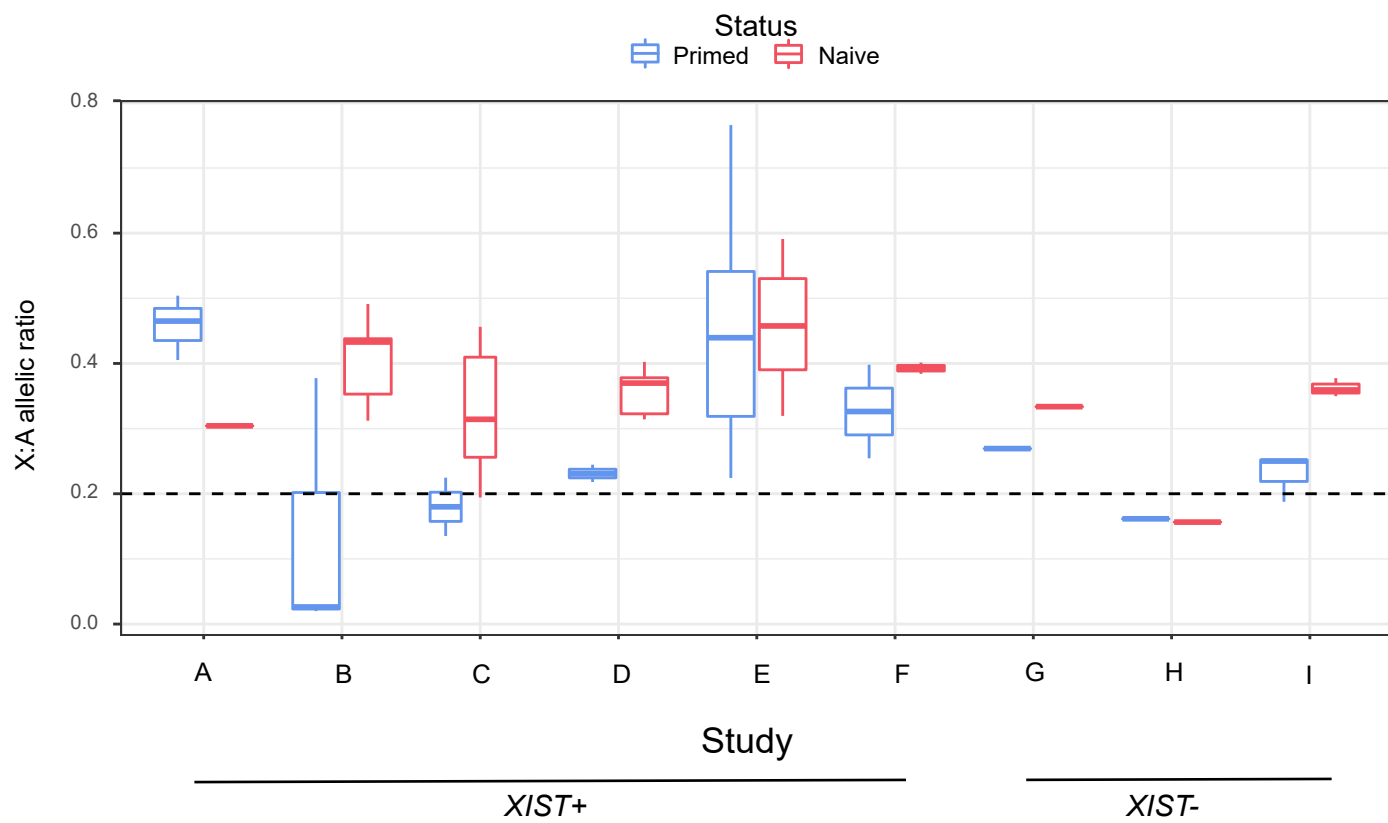

B

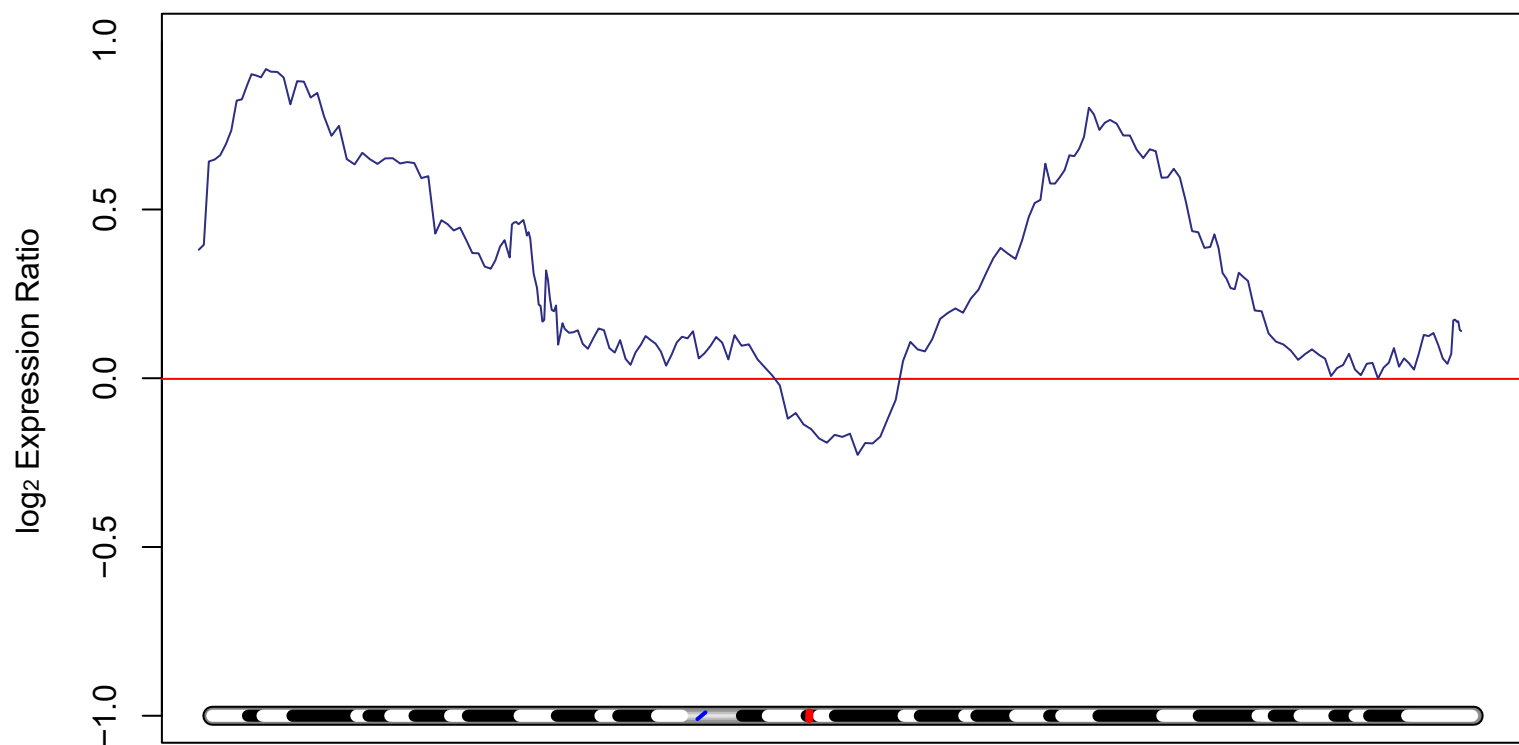

Supplement: Supplementary file 1 [file cells-11-01729-s001.zip › Figure S2 .pdf]

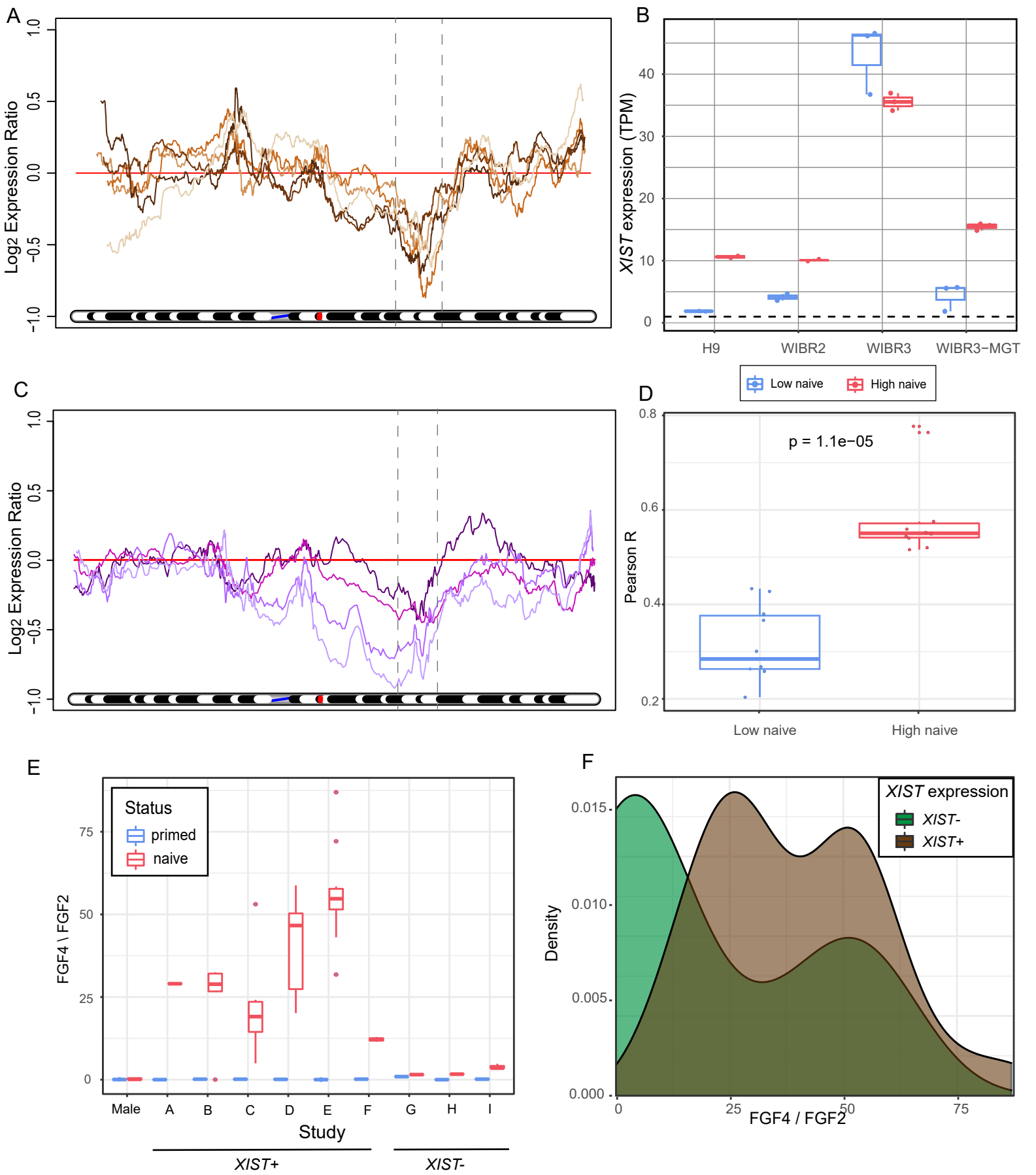

Supplement: Supplementary file 1 [file cells-11-01729-s001.zip › Figure S3.pdf]
